# 6-Methyl-2-pyridyl *N*-acetyl-1-thio-β-d-glucosa­minide methanol monosolvate

**DOI:** 10.1107/S1600536810036238

**Published:** 2010-09-15

**Authors:** Bo Chen, Miao Guo, Wei-Hua Jin, Yan-Wei Wang, Hong-Ze Liang

**Affiliations:** a Faculty of Materials Science and Chemical Engineering, Ningbo University, Zhejiang 315211, People’s Republic of China

## Abstract

In the title compound, C_14_H_20_N_2_O_5_S·CH_4_O, the pyran­ose and pyridine rings are linked through an S atom. The pyran­ose ring has a normal chair conformation. An intra­molecular O—H⋯N hydrogen bond occurs. Inter­molecular O—H⋯O, N—H⋯O, O—H⋯N and weak C—H⋯O hydrogen bonding is present in the crystal structure.

## Related literature

For applications of glucopyran­osides, see: Ashry *et al.* (2006 ▶). For the structure of an α-d-glucosa­minide, see: Harrison *et al.* (2007 ▶).
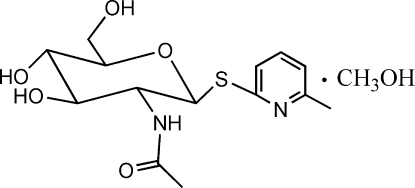


## Experimental

### 

#### Crystal data

C_14_H_20_N_2_O_5_S·CH_4_O
*M* *_r_* = 360.42
Orthorhombic, 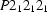

*a* = 7.3841 (15) Å
*b* = 14.041 (3) Å
*c* = 17.038 (4) Å
*V* = 1766.5 (6) Å^3^
*Z* = 4
Mo *K*α radiation
μ = 0.22 mm^−1^
*T* = 296 K
0.51 × 0.27 × 0.2 mm

#### Data collection

Bruker SMART CCD area-detector diffractometer
Absorption correction: multi-scan (*SADABS*; Bruker, 2001 ▶) *T*_min_ = 0.932, *T*_max_ = 0.950
12687 measured reflections
3173 independent reflections
2997 reflections with *I* > 2σ(*I*)
*R*_int_ = 0.161

#### Refinement

*R*[*F* ^2^ > 2σ(*F* ^2^)] = 0.066
*wR*(*F* ^2^) = 0.170
*S* = 1.05
3173 reflections
222 parameters
H-atom parameters constrained
Δρ_max_ = 0.56 e Å^−3^
Δρ_min_ = −0.70 e Å^−3^
Absolute structure: Flack (1983 ▶), 1334 Fiedel pairs
Flack parameter: 0.01 (12)

### 

Data collection: *SMART* (Bruker, 2007 ▶); cell refinement: *SAINT* (Bruker, 2007 ▶); data reduction: *SAINT*; program(s) used to solve structure: *SHELXTL* (Sheldrick, 2008 ▶); program(s) used to refine structure: *SHELXTL*; molecular graphics: *SHELXTL*; software used to prepare material for publication: *SHELXTL*.

## Supplementary Material

Crystal structure: contains datablocks I, global. DOI: 10.1107/S1600536810036238/xu5024sup1.cif

Structure factors: contains datablocks I. DOI: 10.1107/S1600536810036238/xu5024Isup2.hkl

Additional supplementary materials:  crystallographic information; 3D view; checkCIF report

## Figures and Tables

**Table 1:** Hydrogen-bond geometry (Å, °)

*D*—H⋯*A*	*D*—H	H⋯*A*	*D*⋯*A*	*D*—H⋯*A*
N1—H1*A*⋯O2^i^	0.86	2.15	2.925 (4)	149
O2—H2*A*⋯O3^ii^	0.82	2.02	2.794 (3)	156
O3—H3*A*⋯O4^ii^	0.82	1.88	2.646 (3)	155
O4—H4*A*⋯O6^iii^	0.82	1.82	2.637 (4)	176
O6—H6⋯N2	0.82	1.98	2.795 (4)	175
C8—H8*A*⋯O5^iv^	0.93	2.48	3.329 (4)	151
C12—H12*C*⋯O1^v^	0.96	2.58	3.520 (4)	165
C15—H15*C*⋯O3^vi^	0.96	2.56	3.367 (5)	142

Symmetry codes: (i) 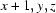; (ii) 
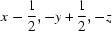
; (iii) 
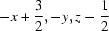
; (iv) 
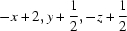
; (v) 
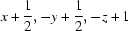
; (vi) 
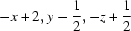
.

**Fig. 1.**
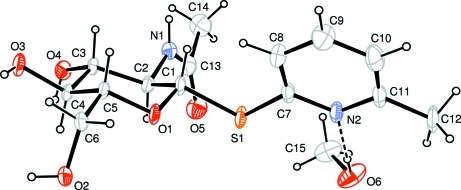
A view of (I) showing the labeling of the non-H atoms and 50% probability ellipsoids. Dashed line indicates the hydrogen bonding.

## supplementary crystallographic information

### Comment

Thioglycosides are widely employed as biological inhibitors, glycosyl donors and enzyme resistant ligands for affinity chromatography (Ashry *et al.*, 2006). Here we report the crystal structure of the title compound (Scheme 1). The title compound crystallizes exclusively as the β anomer. The molecule contains a pyranose ring and a pyridine ring linked by a sulfur atom. The pyranose ring has a normal chair conformation, similar to that found in an α-D-glucosaminide (Harrison *et al.* 2007). The extensive hydrogen bonding network is present in the crystal structure, involving O—H···O, O—H···N and N—H···O hydrogen bonding (Table 1). Weak intermolecular C—H···O hydrogen bonding is also present in the crystal structure.

### Experimental

6'-Methyl-2'-pyridyl-2,3,4,6-tetraacetyl-1-thio-β-D-glucosaminide (1.5 g, 3.3 mmol) was dissolved in MeOH (10 ml) and one equivalent MeONa was added. The process of deacetylation was monitored by ^1^H NMR. After removal of the solvent, the solid residue was washed with ethanol and ether, and then crystallized from H_2_O/MeOH to give the title compound (0.23 g) as colorless crystals.

### Refinement

H atoms were placed in calculated positions and treated using a riding-model, C–H = 0.93–0.98 Å with U_iso_(H) = 1.2U_eq_(C) or 1.5U_eq_(C), N–H = 0.86 with U_iso_(H) = 1.2U_eq_(N), O—H = 0.82 Å with U_iso_(H) = 1.5U_eq_(O).

### Crystal data

**Table:**

C_14_H_20_N_2_O_5_S·CH_4_O	*F*(000) = 768
*M**_r_* = 360.42	*D*_x_ = 1.355 Mg m^−^^3^
Orthorhombic, *P*2_1_2_1_2_1_	Mo *K*α radiation, λ = 0.71073 Å
Hall symbol: P 2ac 2ab	Cell parameters from 12040 reflections
*a* = 7.3841 (15) Å	θ = 1.9–24.5°
*b* = 14.041 (3) Å	µ = 0.22 mm^−^^1^
*c* = 17.038 (4) Å	*T* = 296 K
*V* = 1766.5 (6) Å^3^	Block, colourless
*Z* = 4	0.51 × 0.27 × 0.2 mm

### Data collection

**Table:**

Bruker SMART CCD area-detector diffractometer	3173 independent reflections
Radiation source: fine-focus sealed tube	2997 reflections with *I* > 2σ(*I*)
graphite	*R*_int_ = 0.161
Detector resolution: 0 pixels mm^-1^	θ_max_ = 25.2°, θ_min_ = 1.9°
φ and ω scans	*h* = −8→8
Absorption correction: multi-scan (*SADABS*; Bruker, 2001)	*k* = −16→16
*T*_min_ = 0.932, *T*_max_ = 0.950	*l* = −20→20
12687 measured reflections	

### Refinement

**Table:**

Refinement on *F*^2^	Secondary atom site location: difference Fourier map
Least-squares matrix: full	Hydrogen site location: inferred from neighbouring sites
*R*[*F*^2^ > 2σ(*F*^2^)] = 0.066	H-atom parameters constrained
*wR*(*F*^2^) = 0.170	*w* = 1/[σ^2^(*F*_o_^2^) + (0.1077*P*)^2^ + 0.760*P*] where *P* = (*F*_o_^2^ + 2*F*_c_^2^)/3
*S* = 1.05	(Δ/σ)_max_ = 0.003
3173 reflections	Δρ_max_ = 0.56 e Å^−^^3^
222 parameters	Δρ_min_ = −0.70 e Å^−^^3^
0 restraints	Absolute structure: Flack (1983), 1334 Fiedel pairs
Primary atom site location: structure-invariant direct methods	Flack parameter: 0.01 (12)

### Special details

**Table:**

Geometry. All esds (except the esd in the dihedral angle between two l.s. planes) are estimated using the full covariance matrix. The cell esds are taken into account individually in the estimation of esds in distances, angles and torsion angles; correlations between esds in cell parameters are only used when they are defined by crystal symmetry. An approximate (isotropic) treatment of cell esds is used for estimating esds involving l.s. planes.
Refinement. Refinement of *F*^2^ against ALL reflections. The weighted *R*-factor *wR* and goodness of fit *S* are based on *F*^2^, conventional *R*-factors *R* are based on *F*, with *F* set to zero for negative *F*^2^. The threshold expression of *F*^2^ > σ(*F*^2^) is used only for calculating *R*-factors(gt) *etc*. and is not relevant to the choice of reflections for refinement. *R*-factors based on *F*^2^ are statistically about twice as large as those based on *F*, and *R*- factors based on ALL data will be even larger.

### Fractional atomic coordinates and isotropic or equivalent isotropic displacement parameters (Å^2^)

**Table:**

	*x*	*y*	*z*	*U*_iso_*/*U*_eq_	
S1	0.80502 (12)	0.14422 (6)	0.34312 (4)	0.0246 (2)	
O1	0.6461 (3)	0.24215 (17)	0.23124 (11)	0.0209 (5)	
O2	0.3258 (3)	0.26631 (17)	0.13049 (12)	0.0238 (5)	
H2A	0.3087	0.2660	0.0829	0.036*	
O3	0.7290 (3)	0.28865 (18)	0.02141 (12)	0.0243 (5)	
H3A	0.6376	0.3204	0.0135	0.036*	
O4	0.9501 (3)	0.12909 (17)	0.04864 (13)	0.0237 (5)	
H4A	0.8695	0.0906	0.0392	0.036*	
O5	1.0621 (4)	−0.03011 (17)	0.24234 (16)	0.0327 (6)	
O6	0.8051 (4)	−0.00609 (19)	0.51072 (18)	0.0449 (8)	
H6	0.7982	0.0517	0.5048	0.067*	
N1	1.0936 (4)	0.1246 (2)	0.20602 (15)	0.0221 (6)	
H1A	1.1689	0.1711	0.2024	0.027*	
N2	0.8034 (4)	0.1914 (2)	0.49004 (14)	0.0232 (6)	
C1	0.8218 (5)	0.2112 (2)	0.25267 (16)	0.0205 (6)	
H1B	0.9010	0.2665	0.2604	0.025*	
C2	0.9047 (4)	0.1441 (2)	0.19007 (17)	0.0191 (6)	
H2C	0.8369	0.0841	0.1891	0.023*	
C3	0.8913 (4)	0.1928 (2)	0.10900 (16)	0.0179 (6)	
H3B	0.9773	0.2460	0.1096	0.021*	
C4	0.7073 (4)	0.2349 (2)	0.09201 (15)	0.0186 (6)	
H4B	0.6190	0.1837	0.0837	0.022*	
C5	0.6461 (4)	0.2985 (2)	0.15999 (16)	0.0189 (6)	
H5A	0.7311	0.3516	0.1658	0.023*	
C6	0.4550 (4)	0.3376 (2)	0.14950 (17)	0.0211 (7)	
H6A	0.4181	0.3690	0.1976	0.025*	
H6B	0.4562	0.3850	0.1081	0.025*	
C7	0.8079 (5)	0.2318 (2)	0.41830 (17)	0.0220 (7)	
C8	0.8187 (5)	0.3293 (2)	0.40626 (18)	0.0272 (7)	
H8A	0.8197	0.3549	0.3559	0.033*	
C9	0.8281 (6)	0.3875 (3)	0.4726 (2)	0.0360 (9)	
H9A	0.8383	0.4532	0.4671	0.043*	
C10	0.8221 (5)	0.3470 (3)	0.5465 (2)	0.0343 (8)	
H10A	0.8265	0.3853	0.5910	0.041*	
C11	0.8095 (5)	0.2491 (3)	0.55384 (17)	0.0268 (7)	
C12	0.8040 (6)	0.2002 (3)	0.63295 (18)	0.0342 (8)	
H12A	0.7956	0.1326	0.6255	0.051*	
H12B	0.7005	0.2221	0.6619	0.051*	
H12C	0.9123	0.2149	0.6616	0.051*	
C13	1.1595 (5)	0.0380 (2)	0.22624 (18)	0.0258 (8)	
C14	1.3633 (5)	0.0325 (3)	0.2284 (2)	0.0370 (9)	
H14A	1.3997	−0.0307	0.2431	0.055*	
H14B	1.4089	0.0773	0.2661	0.055*	
H14C	1.4111	0.0473	0.1775	0.055*	
C15	0.9488 (6)	−0.0426 (3)	0.4639 (3)	0.0419 (10)	
H15A	1.0311	0.0080	0.4511	0.063*	
H15B	0.9002	−0.0691	0.4164	0.063*	
H15C	1.0120	−0.0912	0.4925	0.063*	

### Atomic displacement parameters (Å^2^)

**Table:**

	*U*^11^	*U*^22^	*U*^33^	*U*^12^	*U*^13^	*U*^23^
S1	0.0396 (5)	0.0254 (4)	0.0087 (4)	−0.0002 (4)	−0.0009 (3)	0.0015 (3)
O1	0.0240 (12)	0.0320 (12)	0.0068 (9)	0.0013 (9)	0.0010 (8)	0.0021 (9)
O2	0.0240 (11)	0.0350 (12)	0.0124 (10)	−0.0047 (10)	−0.0019 (9)	0.0012 (9)
O3	0.0243 (12)	0.0387 (14)	0.0100 (10)	0.0057 (11)	0.0008 (8)	0.0050 (9)
O4	0.0247 (11)	0.0313 (12)	0.0150 (10)	0.0001 (10)	0.0048 (9)	−0.0054 (9)
O5	0.0398 (15)	0.0250 (13)	0.0333 (14)	0.0013 (11)	0.0009 (12)	0.0023 (11)
O6	0.0568 (19)	0.0269 (13)	0.0509 (17)	0.0004 (15)	0.0235 (16)	0.0076 (12)
N1	0.0221 (14)	0.0258 (14)	0.0184 (13)	−0.0009 (11)	−0.0029 (11)	0.0024 (11)
N2	0.0258 (14)	0.0322 (15)	0.0117 (12)	0.0010 (13)	−0.0009 (11)	0.0008 (10)
C1	0.0252 (15)	0.0290 (16)	0.0073 (12)	0.0010 (14)	0.0000 (12)	−0.0004 (12)
C2	0.0237 (15)	0.0215 (15)	0.0120 (13)	−0.0002 (13)	0.0007 (11)	0.0014 (12)
C3	0.0224 (15)	0.0246 (16)	0.0068 (13)	−0.0012 (12)	0.0020 (12)	−0.0024 (12)
C4	0.0223 (16)	0.0270 (16)	0.0064 (13)	−0.0029 (13)	0.0026 (11)	−0.0002 (11)
C5	0.0271 (16)	0.0219 (14)	0.0078 (13)	−0.0031 (13)	0.0002 (11)	0.0009 (12)
C6	0.0238 (16)	0.0288 (16)	0.0107 (13)	−0.0020 (13)	−0.0010 (12)	−0.0013 (12)
C7	0.0218 (15)	0.0331 (17)	0.0111 (13)	0.0014 (15)	0.0003 (12)	−0.0026 (12)
C8	0.0349 (18)	0.0301 (17)	0.0167 (15)	0.0039 (15)	0.0015 (15)	0.0009 (12)
C9	0.045 (2)	0.0299 (18)	0.0335 (19)	0.0033 (17)	0.0035 (18)	−0.0029 (15)
C10	0.0397 (19)	0.041 (2)	0.0221 (16)	0.0042 (18)	0.0008 (16)	−0.0115 (15)
C11	0.0231 (15)	0.045 (2)	0.0124 (14)	0.0043 (15)	−0.0010 (13)	−0.0036 (14)
C12	0.042 (2)	0.052 (2)	0.0090 (14)	0.0022 (19)	−0.0021 (15)	−0.0020 (14)
C13	0.039 (2)	0.0228 (16)	0.0155 (14)	0.0028 (15)	−0.0014 (14)	−0.0017 (12)
C14	0.036 (2)	0.034 (2)	0.041 (2)	0.0059 (16)	−0.0039 (17)	0.0026 (16)
C15	0.035 (2)	0.037 (2)	0.054 (3)	0.0018 (18)	0.009 (2)	0.0094 (19)

### Geometric parameters (Å, °)

**Table:**

S1—C7	1.776 (3)	C4—C5	1.531 (4)
S1—C1	1.810 (3)	C4—H4B	0.9800
O1—C1	1.416 (4)	C5—C6	1.524 (4)
O1—C5	1.449 (3)	C5—H5A	0.9800
O2—C6	1.420 (4)	C6—H6A	0.9700
O2—H2A	0.8200	C6—H6B	0.9700
O3—C4	1.429 (3)	C7—C8	1.386 (5)
O3—H3A	0.8200	C8—C9	1.397 (5)
O4—C3	1.431 (4)	C8—H8A	0.9300
O4—H4A	0.8200	C9—C10	1.382 (5)
O5—C13	1.227 (4)	C9—H9A	0.9300
O6—C15	1.423 (5)	C10—C11	1.383 (5)
O6—H6	0.8200	C10—H10A	0.9300
N1—C13	1.355 (4)	C11—C12	1.513 (4)
N1—C2	1.447 (4)	C12—H12A	0.9600
N1—H1A	0.8600	C12—H12B	0.9600
N2—C7	1.348 (4)	C12—H12C	0.9600
N2—C11	1.357 (4)	C13—C14	1.507 (6)
C1—C2	1.550 (4)	C14—H14A	0.9600
C1—H1B	0.9800	C14—H14B	0.9600
C2—C3	1.545 (4)	C14—H14C	0.9600
C2—H2C	0.9800	C15—H15A	0.9600
C3—C4	1.510 (5)	C15—H15B	0.9600
C3—H3B	0.9800	C15—H15C	0.9600
			
C7—S1—C1	104.68 (15)	O2—C6—H6A	108.9
C1—O1—C5	112.5 (2)	C5—C6—H6A	108.9
C6—O2—H2A	109.5	O2—C6—H6B	108.9
C4—O3—H3A	109.5	C5—C6—H6B	108.9
C3—O4—H4A	109.5	H6A—C6—H6B	107.7
C15—O6—H6	109.5	N2—C7—C8	123.4 (3)
C13—N1—C2	124.3 (3)	N2—C7—S1	111.3 (2)
C13—N1—H1A	117.9	C8—C7—S1	125.3 (2)
C2—N1—H1A	117.9	C7—C8—C9	117.5 (3)
C7—N2—C11	118.3 (3)	C7—C8—H8A	121.3
O1—C1—C2	111.8 (2)	C9—C8—H8A	121.3
O1—C1—S1	108.4 (2)	C10—C9—C8	119.6 (3)
C2—C1—S1	107.3 (2)	C10—C9—H9A	120.2
O1—C1—H1B	109.8	C8—C9—H9A	120.2
C2—C1—H1B	109.8	C9—C10—C11	119.5 (3)
S1—C1—H1B	109.8	C9—C10—H10A	120.2
N1—C2—C3	108.2 (2)	C11—C10—H10A	120.2
N1—C2—C1	111.5 (3)	N2—C11—C10	121.6 (3)
C3—C2—C1	108.7 (2)	N2—C11—C12	116.2 (3)
N1—C2—H2C	109.5	C10—C11—C12	122.2 (3)
C3—C2—H2C	109.5	C11—C12—H12A	109.5
C1—C2—H2C	109.5	C11—C12—H12B	109.5
O4—C3—C4	112.3 (2)	H12A—C12—H12B	109.5
O4—C3—C2	110.2 (2)	C11—C12—H12C	109.5
C4—C3—C2	113.7 (2)	H12A—C12—H12C	109.5
O4—C3—H3B	106.7	H12B—C12—H12C	109.5
C4—C3—H3B	106.7	O5—C13—N1	123.1 (3)
C2—C3—H3B	106.7	O5—C13—C14	122.6 (3)
O3—C4—C3	105.5 (2)	N1—C13—C14	114.3 (3)
O3—C4—C5	111.2 (2)	C13—C14—H14A	109.5
C3—C4—C5	110.4 (2)	C13—C14—H14B	109.5
O3—C4—H4B	109.9	H14A—C14—H14B	109.5
C3—C4—H4B	109.9	C13—C14—H14C	109.5
C5—C4—H4B	109.9	H14A—C14—H14C	109.5
O1—C5—C6	107.1 (2)	H14B—C14—H14C	109.5
O1—C5—C4	108.3 (2)	O6—C15—H15A	109.5
C6—C5—C4	113.2 (3)	O6—C15—H15B	109.5
O1—C5—H5A	109.3	H15A—C15—H15B	109.5
C6—C5—H5A	109.3	O6—C15—H15C	109.5
C4—C5—H5A	109.3	H15A—C15—H15C	109.5
O2—C6—C5	113.3 (3)	H15B—C15—H15C	109.5

### Hydrogen-bond geometry (Å, °)

**Table:**

*D*—H···*A*	*D*—H	H···*A*	*D*···*A*	*D*—H···*A*
N1—H1A···O2^i^	0.86	2.15	2.925 (4)	149
O2—H2A···O3^ii^	0.82	2.02	2.794 (3)	156
O3—H3A···O4^ii^	0.82	1.88	2.646 (3)	155
O4—H4A···O6^iii^	0.82	1.82	2.637 (4)	176
O6—H6···N2	0.82	1.98	2.795 (4)	175
C8—H8A···O5^iv^	0.93	2.48	3.329 (4)	151
C12—H12C···O1^v^	0.96	2.58	3.520 (4)	165
C15—H15C···O3^vi^	0.96	2.56	3.367 (5)	142

Symmetry codes: (i) *x*+1, *y*, *z*; (ii) *x*−1/2, −*y*+1/2, −*z*; (iii) −*x*+3/2, −*y*, *z*−1/2; (iv) −*x*+2, *y*+1/2, −*z*+1/2; (v) *x*+1/2, −*y*+1/2, −*z*+1; (vi) −*x*+2, *y*−1/2, −*z*+1/2.
